# Fragmentation in health system financing: a major challenge for improving effectiveness, efficiency, equity and choice within health systems

**DOI:** 10.1136/bmjph-2025-004716

**Published:** 2026-10-05

**Authors:** Thomas Hone, Matias Mrejen, Pedro P Barros, Rudi Rocha, Rodrigo Moreno-Serra, Diana Pinto, Octavio Gómez-Dantés, Edson Serván-Mori, Laura Flamand, Kevin Croke, Peter Berman, Thomas J Bossert

**Affiliations:** 1 Public Health Policy Evaluation Unit, Imperial College London, London, UK; 2 Instituto de Estudos para Políticas de Saúde (IEPS), Sao Paulo, Brazil; 3 Instituto René Rachou, Fundação Oswaldo Cruz, Fiocruz, Belo Horizonte, Brazil; 4 Nova School of Business and Economics, Lisbon, Portugal; 5 Sao Paulo School of Business Administration (FGV EAESP), Sao Paulo, Brazil; 6 Centre for Health Economics, University of York, York, UK; 7 Universidad Nacional de Colombia, Bogotá, Colombia; 8 Center for Health Systems Research, National Institute of Public Health, Cuernavaca, Mexico; 9 Center for International Studies, College of Mexico AC, Mexico City, Mexico; 10 Department of Global Health and Population, Harvard T H Chan School of Public Health, Boston, Massachusetts, USA

**Keywords:** economics, Scoping Review, Health Services Accessibility

## Abstract

Health system fragmentation is the division and lack of coordination among major health system functions such as financing, service delivery or governance. It is often claimed that fragmentation significantly affects the efficiency, equity and quality, often leading to inefficiencies and poor performance. However, the fragmentation of health systems, particularly from the structural and financing perspective, is conceptually underexplored. This article focuses on fragmentation of health system financing, a critical driver of health system performance and presents a framework and narrative literature review of the impacts of health system financing fragmentation on health system performance. Health system financing fragmentation is explored across six domains (purchasers, risk pools, eligibility categories, benefit packages, providers and payments) and the impacts of such fragmentation are analysed.

Existing evidence, mostly from high-income settings and a few low- and middle-income country (LMIC) case studies, indicates that highly fragmented health system financing can lead to duplication of services, high administrative costs and inequitable access to care. However, empirical evidence is limited, context-specific and rarely from LMICs. Where comparative empirical studies are available, they generally find that health systems with fewer, more coordinated purchasers and risk pools tend to perform better in terms of equity and efficiency. Conversely, case-study evidence and descriptive analyses suggest that systems with multiple, uncoordinated eligibility categories and benefits packages often face challenges in achieving universal health coverage and equitable access to high-quality care. Competition, lack of regulation, complex payment systems and decentralisation have been found to further contribute to health system fragmentation. While economic theory highlights that some level of fragmentation and competition may enhance responsiveness and innovation, the empirical support for these potential benefits is weak and highly context-dependent. Overall, the balance of available evidence suggests that, in most settings, excessive financing fragmentation tends to undermine the health system goals of efficiency and equity.

**Summary box**

- Despite being a pervasive challenge for health system performance, there is relatively weak and uneven comparative empirical evidence on how health system financing fragmentation drives overall performance. Some country-comparison studies and systematic reviews find associations suggesting that fragmentation can compromise the health system goals of promoting equity, financial risk protection and improving health. However, other studies show there may be some benefits of fragmentation including from increased competition and service flexibility and adaptation, although these findings are typically context-specific and often based on single-country or programme case studies.
- The separate domains of fragmentation appear to be interconnected and there is a systemic interdependence across domains; high levels of fragmentation across multiple domains likely multiply, exacerbating challenges in meeting health system goals. Addressing fragmentation in one domain, without addressing other domains, is unlikely to substantially reduce overall system fragmentation. This suggests that comprehensive and holistic approaches to addressing financing fragmentation across multiple domains are needed.
- Decentralised health systems pose further challenges and may increase system fragmentation. Regulation and strategic coordination are likely to be vital policy levers for addressing the negative effects of fragmentation, for example, through consolidation of risk pools, harmonisation of benefits packages and fiscal coordination.
- Further empirical and comparative research, particularly from low- and middle-income countries, is needed to better understand the impacts of health system financing fragmentation and disentangle the trade-offs across fragmentation domains in diverse health system contexts, moving beyond theoretical expectations and single-case descriptions towards more robust comparative designs.

## Introduction

 Health system fragmentation is a major determinant of how health systems function, including their ability to deliver accessible, efficient and high-quality healthcare, improve health, guarantee responsiveness and protect people from financial hardship.^1–4^ The term ‘fragmentation’ has been used to describe multiple facets of health systems, typically linked to inefficiencies and poor performance, such as duplication and administrative costs,^5^ care coordination,^6^ service continuity,^7,8^ care across different providers,^9,10^ data sharing,^11^ patient pathways^12,13^ and financing arrangements.^14^ Fragmentation contributes to inequalities, as low-income populations often face limited access, poorer quality care, delayed diagnosis and exploitation by unregulated private providers.^5,15–17^ The COVID-19 pandemic highlighted fragmentation as a barrier to pandemic preparedness and response—partially due to the uncoordinated actions of public health agencies.^18^ However, there is no clear consensus on what health system fragmentation entails or on how it affects health system performance across contexts.

Despite a growing body of work on integrated care and service-delivery fragmentation, there is comparatively little conceptual and empirical work that focuses specifically on fragmentation in health system financing. Existing studies often examine single dimensions, such as purchaser competition, risk pooling, benefit packages or payment mechanisms, mostly in isolation and evidence is concentrated in high-income settings. There is a lack of a unified framework that links these dimensions to health system goals. As a result, policymakers lack a structured way to diagnose where financing fragmentation arises, to understand how different domains interact, and to compare the likely consequences of alternative reform options,

This paper addresses this gap by providing a framework for health system financing fragmentation, clarifies definitions and measurement approaches, and presents a narrative review of the literature on the impact of specific aspects of fragmentation. The paper focuses on fragmentation in the financing of health systems and its implications for overall system performance and the achievement of health system goals. While other complementary efforts are vital to improve the organisational design and management of health systems (eg, governance, resourcing, information, training, logistics), we argue health system financing is a critical driver of overall health system performance. First, political and societal choices around raising and spending funds shape health system structure and governance. Second, financial flows are powerful determinants of downstream activities and service performance,^19^ because how money is distributed, the conditions attached to this funding and the total funds available determine what resources and services can and cannot be provided. Third, we distinguish our focus from service integration literature, which relates more to clinical service delivery and is arguably better addressed at the local level.

## Methodology

We undertook a narrative (conceptual) review of the literature to (1) identify existing frameworks and theories relevant to health system financing fragmentation (eg, competition, market dynamics) and (2) summarise empirical evidence on the impacts of specific domains of fragmentation (eg, number of purchasers). The aim was to map key concepts, mechanisms and illustrative evidence rather than to conduct a fully systematic or scoping review.

We searched PubMed, Ovid (including MEDLINE), and Google Scholar between March 2023 and April 2024. We used combinations of key terms related to fragmentation—for example, “multiple benefits packages”, “uncoordinated risk pools”, “provider competition” and “fragmented payment mechanisms”. The references from relevant papers and seminal reports (eg, from WHO and World Bank) were screened for additional useful literature and the authors supplemented the analysis with literature from their own knowledge and expertise due to the paucity of empirical evidence on financing fragmentation.

We included publications that: (1) discussed conceptualisations or frameworks of health system financing and fragmentation, or related concepts and theories or (2) provided empirical or descriptive evidence relating any of six financing domains (purchasers, risk pools, eligibility categories, benefit packages, providers, payments) to health system goals such as equity, efficiency, financial protection or quality. We considered peer-reviewed journal articles, books and relevant technical reports in English. We did not apply formal study-design restrictions. We prioritised studies that offered comparative analyses (eg, cross-country comparisons, quasi-experimental evaluations or systematic reviews) and those that clearly linked fragmentation in a specific domain to health system goals. Context-specific case studies and descriptive analyses were included where they provided important insights into mechanisms, particularly from low- and middle-income countries (LMICs).

The paper is organised as follows. We first explore frameworks and literature related to health systems financing, the definition of fragmentation and key relevant economic theories. We selected a suitable framework based on relevance to the topic, that is, being specific to financing and aligning with key domains and relationships we wished to unpack. We used relevant economic theory and studies to outline key background on public and private healthcare markets and the relationships that govern these. Second, we discuss each of the domains of financing fragmentation, outlining key definitions and options for measurement. We summarise key literature on the impacts of each of the domains on fragmentation on health systems goals, attempting to draw on diverse literature from across countries and settings. Where possible, we illustrate our arguments using examples from specific countries, reforms and policies.

### Patient and public involvement

Patients and the public were not involved in this study.

## Frameworks for health system financing, definitions and key theories

### A health systems framework for conceptualising health system financing fragmentation

We use a health system financing framework by Kutzin *et al* to frame our discussion (figure 1), which is based on previous health system frameworks,^20,21^ including Murray and Frenk.^22^ Other frameworks exist,^20,21^ but we choose this one because it is highly aligned with and is informed by, other frameworks,^20,21,23^ it outlines functions, intermediate objectives and health system goals and focuses on health system financing. The framework considers four functions of the health system that policy-makers can influence to affect system performance^4^—stewardship/governance, resource generation, service delivery and financing. Stewardship/governance includes key subfunctions such as system design, legislation, strategic planning, regulation, performance assessment, priority setting, intersectoral advocacy and consumer protection. Regulation is a key subfunction (under stewardship/governance), and as will be argued below, has important implications for system fragmentation. Resource generation relates to health professionals, facilities, medicines, equipment and information technology, while the service delivery function covers the provision of clinical and public health services. Lastly, the financing subfunction covers how funds are collected and pooled, and how these funds are used to purchase services and deliver different benefits to populations.

**Figure 1:**
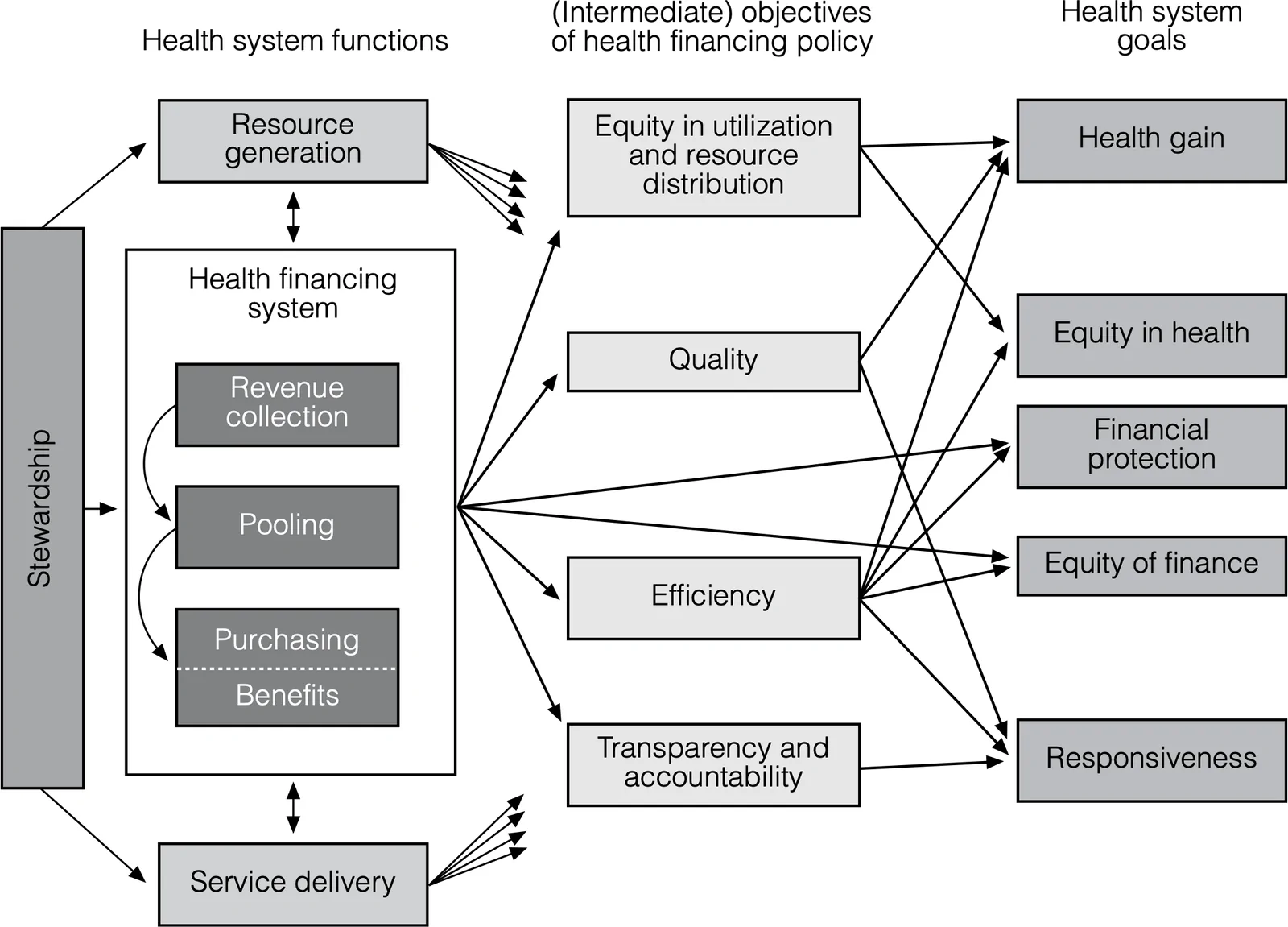
A health system framework with a focus on health system financing. Source: Kutzin J, Cashin C, and Jakab M 2010 ^24^

This framework outlines how health system functions affect intermediate objectives and the final (or intrinsic) goals of a health system. The intermediate objectives are desirable attributes of health systems (and the services used by patients) and are important for overall health system goals. Intermediate objectives in health system financing include equity in utilisation and resource distribution, service quality, system efficiency and transparency and accountability. Health system goals are the overarching objectives that health systems aim to deliver. These include improved health, financial risk protection, responsiveness (and satisfaction) and equity.^20,24^

### Defining health system fragmentation

Health system fragmentation can be defined as the division, without coordination, of health system functions.^25^ Fragmentation is generally discussed in relation to health system financing (the focus on this paper) or service delivery (covered by the wide body of literature on integrated care). In the simplest terms, fragmentation can be measured quantitatively through the number of separate elements (eg, agents, pools, funds), with higher numbers indicative of greater fragmentation. Because more elements exist, the complexity in coordinating these elements increases. Other measures of fragmentation may consider the relative size and power of these separate elements.

A lack of coordination is a key aspect of health system fragmentation. Coordination is defined as the organisation of elements within a system for harmonious functioning and effective achievement of desired goals.^26^ Therefore, the problem of fragmented health systems financing is principally the increased number of uncoordinated elements, which make achieving health system goals (both intermediate and final goals) more difficult (figure 1). Three additional themes will also be drawn on throughout, which both affect coordination and fragmentation and are not independently orientated towards the same goals: (1) decentralisation—the transfer of control, power and decision-making away from central to local bodies, non-governmental organisations or private entities^27^; (2) regulation—the laws, rules, policies and principles that aim to govern and direct health systems to better achieve health system goals and (3) competition—how different purchasers and providers compete over price and quality.

### Market and non-market theories relating to health systems

The mechanisms and behaviours underpinning health systems are important for health system fragmentation. In nearly all countries, both public and private systems operate. Private healthcare is driven by market dynamics; prices are influenced by competition among insurers and patient demand and individuals choose from multiple providers. Supply and demand dynamics underpin this free-market model, yet effective functioning relies on several key assumptions. Competition must be ‘perfect’ where numerous buyers and sellers interact, and no single entity can influence prices. Buyers must be rational and able to choose the best product at the lowest price, which requires symmetrical information and informed decision-making by both buyers and sellers. Effective and efficient markets also require no externalities (eg, environmental damage, infectious diseases). However, the health system goals (health outcomes, satisfaction and financial risk protection) that effective and efficient healthcare markets can be measured against are difficult to optimise simultaneously and require deliberate trade-offs in policy design.

Healthcare markets can be composed of three related markets or transactions: (1) the health insurance market, between patients and the insurer; (2) the healthcare purchasing market involving insurers and healthcare providers and (3) the healthcare provision market, between patients and providers (figure 2).^28^

**Figure 2:**
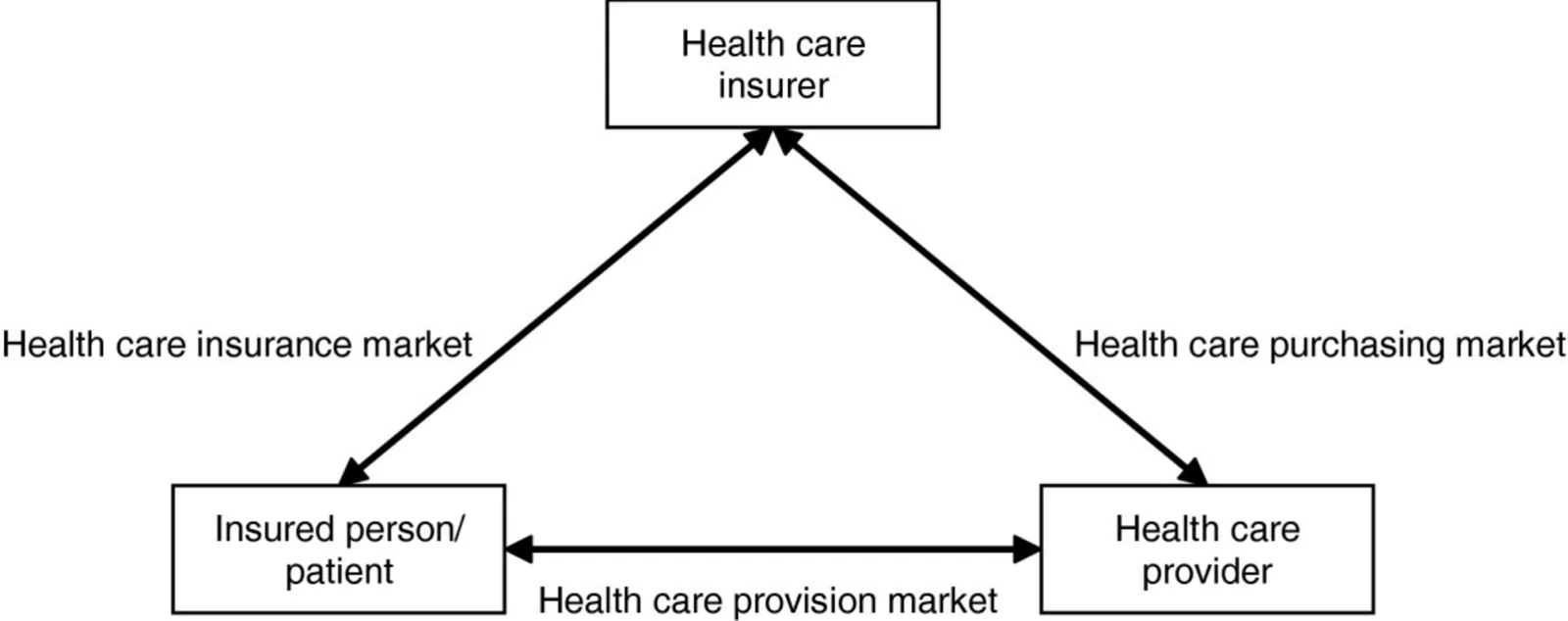
Framework of healthcare insurance markets. Source: Victoor *et al*.^97^

Free-market assumptions rarely hold in healthcare markets leading to inefficient resource allocation and suboptimal health outcomes. These failures necessitate regulation and government intervention. Patients typically lack the medical knowledge providers possess, creating information asymmetry. Externalities, both positive and negative, are often unaccounted for and high barriers to entry and exit limit competition. Monopoly power can emerge, driving up costs and restricting access. Markets are also prone to moral hazard, where individuals overuse care because they are insulated from costs, and adverse selection, where high-risk individuals disproportionately seek insurance. Access to private care depends on ability to pay, leaving uninsured low-income populations to forgo care or face catastrophic expenses. To address these issues, many systems replace direct patient–provider transactions with contracts, commissioning and procurement mechanisms. These arrangements enable purchasers to set quality and pricing standards and align service delivery with health system goals, reducing some inefficiencies and inequities of unregulated markets.

Government-funded healthcare systems are typically funded from taxes or through national insurance schemes and delivered through public organisations, relatively insulated from free-market dynamics. Ministries or public agencies usually make organisational and resourcing decisions prioritising equitable access based on need rather than ability to pay. However, these systems face challenges like private healthcare markets that jeopardise efficient and effective healthcare provision. Misallocated resources may stem from bureaucratic inefficiencies or political considerations, leading to waste and suboptimal use of funds. Excessive or poorly designed regulations may stifle innovation, reduce efficiency and increase costs. Access and choice may be limited, particularly where long wait times or service shortages exist. Resource constraints and weak incentives for performance may compromise care quality. Corruption and mismanagement within both public and private healthcare systems can also lead to inequitable access and poor service delivery. Non-profit organisations may play significant roles in some parts of the health system operating without direct government control or subject to market dynamics.

In nearly all countries, public and private systems do not operate in isolation. Private healthcare markets rarely operate without government regulation or intervention, while public health systems are influenced (either inadvertently or intentionally) by market forces. Even in systems with minimal market dynamics, public choice theory shows that economic and political incentives shape policymaker behaviour, which may not always align with overall health system goals.^29^

Most countries adopt hybrid models that combine public financing with elements of regulated competition to achieve health system objectives. Regulation plays a central role in structuring these arrangements. For example, private markets are often regulated to limit risk selection and ensure minimum coverage, while clinical guidelines aim to reduce the negative effects of information asymmetry. Public systems may introduce purchaser–provider splits to create quasi-markets (a healthcare purchasing market (figure 2)) and allow patient choice to incentivise quality and efficiency. These mechanisms are typically implemented while preserving universal access and coverage, often through tax-based or national insurance schemes, to prevent financial hardship. Blending the principles of health insurance markets and public healthcare gives rise to situations of multiple payers (ie, multiple insurers including governments) and single payers with multiple providers (eg, governments or national insurance agencies, and public and private providers in competition for contracts). Here the links with health system fragmentation become more evident. These situations can be made more complex in large, decentralised systems where subnational agencies and governments operate and interact with local healthcare provision markets. Hence, many large countries develop national institutions to promote coordination, for example, the National Primary Health Care Development Agency in Nigeria and Ayushman Bharat institutions in India.

These market and non-market dynamics are closely linked to health system financing fragmentation. First, we can expand our ‘static’ conceptualisation of the health system financing function (figure 1) incorporating market dynamics which are based on transactions, motivations and regulation. Second, we conceptualise the key agents in the financing of the health system as patients, providers and insurers (governments can be both national insurers and/or providers) and examine fragmentation in relation to each. Third, we analyse fragmentation across financing functions (revenue collection, risk-pooling, benefits, purchasing, payments) in relation to these agents and their interactions with each other and health system goals. We draw on empirical evidence and syntheses to substantiate our arguments. However, nearly all studies come from high-income countries, particularly Europe and North America. Lastly, we consider how these agents operate across the financing functions in the wider context of competition and regulation, and their relationship with fragmentation.

## Conceptualising fragmentation and its impacts

The fragmentation of health system financing has previously been conceptualised into multiple domains.^25^ We posit that there are six key domains for examining financial fragmentation of health systems—purchasers, risk pooling, eligibility categories, benefits packages, payments and providers (table 1). We also propose some illustrative examples of potential indicators for each of the fragmentation domains (table 2).

**Table 1:** Six domains of health systems financing fragmentation

Domain	Measure	Low fragmentation example	High fragmentation example
Purchaser fragmentation	Number and asymmetry of purchasers operating in a health system and the extent to which they coordinate their behaviours and what they pay.	Government purchaser or large (eg, national or state-level) insurance agency, or multiple government agencies/insurers acting as coordinated purchaser. For example, Costa Rica, Denmark, Norway, Singapore, Sweden, Turkey, UK.	Multiple insurers and/or government agencies operating without coordination, or high private levels of healthcare purchasing. For example, Switzerland, Netherlands, Ireland, Germany.
Risk-pooling fragmentation	Number and size of distinct risk pools (ie, with limited cross-subsidisation) within a health system.	National or large, consolidated risk pooling systems or separate pools with high degree of cross-subsidisation. For example, Belgium, Germany, Israel, the Netherlands, Switzerland.	Numerous small risk pools, or systems without large insurance pools and high levels of OOP and fee-for-service. For example, India, Nigeria, Pakistan, Bangladesh, Ethiopia.
Eligibility fragmentation	Number of different eligibility categories within a health system.	Few separate eligibility categories, or universalist systems. For example, Sweden, Norway, Denmark, UK.	Population segmented into multiple distinct groups based on characteristics (eg, employment). For example, USA (Medicare, Medicaid, employer-provided insurance, individually purchased insurance), Mexico (IMSS, ISSSTE, IMSS-Bienestar), Colombia, Argentina, India (state-level schemes).
Benefit package fragmentation	The number, comprehensiveness and coverage of distinct benefits packages.	Few distinct benefits packages with high levels of coverage and comprehensive coverage of services. Examples: high-income group health insurance in Germany (different from the benefits under sickness funds to the rest of the population)	Numerous distinct benefit packages with low levels of population coverage and/or many services remaining uncovered. US Medicaid variations across states; Korea, Chile (in private sector), Mexico (social security agencies and IMSS-Bienestar). Brazil (SUS and health private insurance plans).
Provider fragmentation	Number of providers operating in a healthcare market and extent to which they can compete on price and/or quality.	Few providers operating without competition. For example, UK NHS, Denmark, Norway, Sweden, Cuba.	Numerous providers operating without coordination and may be in competition. For example, Chile, Colombia, Mexico, India, USA.
Payment fragmentation	Number of payment mechanisms and the extent to which they affect coordination of the system.	Fewer, streamlined payment mechanisms that are coordinated, for example, UK, Germany.	Numerous mechanisms without coordination, likely offering contradicting incentives, for example, Brazil, Colombia, Indonesia.

IMSS, Instituto Mexicano del Seguro Social;; ISSTE, Instituto de Seguridad y Servicios Sociales de los Trabajadores del Estado; NHS, National Health Service; OOP, out-of-pocket; SUS, Sistema Único de Saúde.

**Table 2:** Six domains of health systems financing fragmentation

Domain	Example indicators
Purchaser fragmentation	Number of purchasers per 10 000 population. Share of public expenditure in total health expenditure. Share of OOP in total health expenditure. Number of health insurance companies per 10 000 population. Purchaser concentration index (eg, Herfindahl-Hirschman Index (HHI)).
Risk-pooling fragmentation	Number of distinct risk pools per 10 000 population. Share of population in the largest risk pool. Medical loss ratio. Distribution of pool sizes (ie, many small or few large pools). Cross-subsidisation measures.
Eligibility fragmentation	Number of eligibility categories/schemes. Coverage gap (population without coverage). Overlap in eligibility (population with dual coverage). Private insurance coverage rate.
Benefit package fragmentation	Number of major benefit packages. Package comprehensiveness (eg, index comparing included services). Coverage of high cost services.
Provider fragmentation	Number of providers per 10 000 population (public/private; by schemes). Hospital bed concentration (HHI). Health workforce imbalance (ratio of public vs private, or across regions). Continuity of care proxy (eg, proportion of patients seen by same provider).
Payment fragmentation	Number of distinct payment mechanisms used by purchasers. Number of payers per provider. Frequency of tariff revisions. Variation of procedure fees across providers. Share of expenditure under performance-linked payment.

OOP, out-of-pocket.

### Purchaser fragmentation

Purchasers are organisations that provide health financing coverage or insurance to a significant share of the population (ie, the insurers in figure 2); a higher number of purchasers indicates greater purchaser fragmentation.^25^ Literature on single-payer and multiple-payer systems is relevant here.^30^ Systems with low purchaser fragmentation are often tax-funded (eg, Canada, the UK, Nordic countries) or have major national insurance systems (eg, Estonia, Costa Rica, Singapore, Turkey), whereby national governments or insurance agencies are the major purchaser. Conversely, highly fragmented systems feature multiple payers and insurers, likely combinations of public and private, for example, France, Chile, Colombia, Georgia, Germany, Kazakhstan, Switzerland, the Netherlands, Thailand, Poland, Ghana, Rwanda and the USA.

Measuring purchaser fragmentation requires assessing how ‘organisations in the system are buying healthcare, the extent to which they behave in similar ways, and whether they pay the same prices’.^31^ For example, multiple national insurers may be regulated and coordinated to act as a monopsony (ie, a single purchaser), effectively reducing purchaser fragmentation. Other key drivers of purchaser fragmentation include low public spending on health, reliance on donor and aid financing, and the introduction of new stand-alone insurance schemes.^32^

What are the trade-offs between greater or less purchaser fragmentation? There is some evidence that equity and efficiency can be compromised in fragmented systems.^32^ A systematic review in 2018 examined 49 studies on the topic concluding that health systems with fewer payers (less purchaser fragmentation) can be more equitable due to increased access and progressive financing, have larger risk pooling (discussed below) and better purchasing efficiency.^33^ More fragmented systems can be costlier, however, mainly from increased administration costs and adverse selection, but can be less prone to government interference, and provide more choice and options to patients. Evidence from LMICs (Laos, Ghana, Kenya, Tanzania) with vertical disease programmes funded by fragmented purchasers (eg, multiple government agencies, private sector and donors) demonstrates challenges with overall system stewardship, uncoordinated planning and budgeting, service duplication and inefficiencies.^34,35^ South Korea, Turkey, Thailand and Indonesia all merged insurance funds resulting in reduced inequalities in financing and service access, better control of healthcare expenditures and increased strategic purchasing.^36^ Turkey successfully reduced purchaser fragmentation by consolidating multiple public insurance schemes to expand insurance coverage, access and improve health and financial risk protection.^37^ In Ethiopia, the merging of public funding and donor funder streams in 2016 into a single performance fund managed by the Ministry of Health has increased coordination of funding streams and strategic oversight, strengthening national health programmes to deliver health gains.^38^ A few health systems have experimented with increasing competition between insurers (eg, the USA, Chile, Colombia, Israel and the Netherlands), although the impacts on system costs remain uncertain^39–41^; downstream determinants, for example, consolidation of providers and selective contracting, appear important. The associations between purchaser fragmentation and efficiency and quality remain unclear.^42^

### Risk pooling fragmentation

Risk pooling is a core function of health financing policy and entails spreading financial risk across the population, so no individual carries the full burden of paying for healthcare at the moment of use. Health systems with smaller risk pools and decreased sharing of financing across purchasers can be considered more fragmented.^25^ One extreme is where everyone pays out-of-pocket (OOP) (fully fragmented), while the opposite is national risk-pooling (wholly unfragmented). Risk pooling fragmentation is inherently related to purchaser fragmentation, as risk pools are often constructed at the organisation level with each purchaser responsible for their own funds. It is also related to regulation and efforts towards coordination. There may be mechanisms that pool or share health financing across population subgroups and/or across purchasers (eg, a payroll tax revenue that also covers healthcare for individuals not working or in the informal sector) (eg, Netherlands, Thailand, Colombia and Ghana). Conversely, single purchasers can manage multiple risk pools (eg, Medicare in Australia^43^) or central regulation can force multiple insurance funds with risk pools to act as one large risk pool (eg, Germany^44^). Thus, measuring risk pooling fragmentation should account for size of the pool and cross-subsidisation across pools.

What are the impacts of different levels of risk pooling on health system goals? As risk pooling is strongly tied to purchasers, the evidence solely for risk pooling is very unclear.^45^ In more fragmented systems, there can also be risk segmentation that separates populations based on health risks and healthcare costs. This can reduce the potential for moral hazard, but also has large equity implications with the poor and sickest members of society bearing the largest costs. Health systems in Latin America are often segmented based on labour status, with different pools for formal and informal workers, associated with different health benefits. This creates major problems of equity, for example, high OOP across schemes in Mexico.^46^ Empirical evidence associates greater risk pooling with lower OOP payments and decreased likelihoods of catastrophic expenditure.^47^ Thus, less fragmented risk pools can reduce the risk of high healthcare costs for individuals and redistribute resources to the poor.^48^ Highly fragmented risk pools may have administrative complexities, increasing costs, and there may be difficulties in achieving successful risk pooling and financial sustainability.^49,50^ Two examples of highly fragmented risk pools include Nigeria where states have been directed to create their own health insurance schemes and Ethiopia where Community Based Health Insurance operates at the district level. Less fragmented risk pools may also help to control expenditures due to monopsony power and economies of scale,^51^ but also become inefficient due to a lack of incentives.

There has been a trend in many countries to increase risk pooling and reduce fragmentation through a range of options: (1) introducing compulsory or automatic coverage; (2) merging different risk pools; (3) cross-subsidisation of pools and (4) harmonisation across pools.^51^ In general, more unified and less fragmented risk pools are desirable, although political and institutional factors may inhibit a completely unified, single risk-pooling system.^51,52^

### Eligibility fragmentation

Eligibility defines whether an individual qualifies to receive coverage under a particular health financing arrangement (eg, insurer or government health system) and determines their entitlement to services within a defined benefits package. Eligibility criteria may be financial (eg, income thresholds), demographic (eg, age, gender), occupational (eg, formal vs informal sector) or geographic (eg, rural vs urban residence). Targeted programmes often use these criteria to focus on specific populations or health needs, such as maternal health, chronic disease management or disability services.

A greater number of distinct eligibility categories within a health system is indicative of a higher degree of fragmentation.^25^ This is particularly evident in systems where different schemes or agencies serve separate population groups—such as formal sector workers, informal workers, civil servants, armed forces or the poor—each with its own financing mechanisms, benefits and provider networks.^53^ In such systems, eligibility fragmentation often reflects broader segmentation in the health system, and this is reflected in similar levels of fragmentation across other fragmentation domains.

An increased number of eligibility categories has implications for equity and efficiency. From an equity perspective, fragmentation can entrench inequalities in access to care. Segmentation of populations into separate eligibility categories inherently implies either alternative benefits packages, premiums or cost-sharing, or differing providers with likely varying service quality (see below).^53,54^ Individuals with similar health needs may receive different levels of coverage or quality of care depending on their eligibility category. From an efficiency standpoint, multiple eligibility categories often necessitate parallel administrative structures, increasing overhead costs and reducing economies of scale.^55^ In Mexico, segmented systems for populations with and without social security have kept per-capita public spending for the latter systematically lower, despite higher health needs and policy efforts to expand coverage.^56^ Fragmentation may also lead to duplication of services or gaps in coverage, particularly when individuals transition between categories—for example, when ageing out of child coverage or changing employment status. Differentiated eligibility also often implies varying levels of cost-sharing, premiums or OOP payments. This can expose vulnerable populations to financial hardship and limit the redistributive potential of pooled financing. In systems where individuals can pick from multiple categories, there are implications for choice and responsiveness; however, there may be confusion and inefficiencies.

What are the impacts of different eligibility categories on health system goals? There is little empirical evidence that isolates the effects of eligibility fragmentation in health systems, as much is inherently tied to differences in benefits packages or cost-sharing. Furthermore, many health system reforms which do alter eligibility categories simultaneously change cost-sharing, services or benefits packages.

### Benefits package fragmentation

Benefits packages are the set of healthcare services, treatments, medications and interventions available to eligible individuals. They are determined by what purchasers pay for. The availability of multiple, diverse benefit packages can be an indication of more fragmentation. On one hand, this can be beneficial for patient choice, allowing individuals to choose packages which suit their needs and levels of risk. However, in many countries there is limited choice as extensive private markets do not exist or are only for high-income populations, or public systems have little diversity in their offering. Diverse benefits packages may also be reflective of segmented systems (including fragmented purchasers, risk pools and eligibility categories) with associated issues around inequalities in healthcare access to specific services, healthcare quality and financial risk protection (especially if paying for services out of the benefit package). Multiple benefit packages may also reflect efforts to provide local packages based on the health needs and priorities of different areas, which may improve efficiency and effectiveness by ensuring that resources are allocated where they are most needed.^57^

To assess the fragmentation of benefits packages, it is important to measure the comprehensiveness of packages and the overall coverage of the population. Counting the number of packages available is not a useful exercise by itself. Where comprehensiveness of the services is low, then substantial conditions likely remain uncovered with patients either forgoing care or paying OOP. Arguably, the fragmentation of these systems with regards to benefits packages is high as there is weak achievement of health system goals (of improved health and financial risk protection). Similarly, where a sizeable proportion of population remain uncovered (eg, if comprehensive packages are available), fragmentation is also high. In situations where packages are comprehensive and coverage is high, we can consider fragmentation lower (although there may be implications for equity due to package diversity) and would be lower still where there is little variability between these packages in the services coverage. At this level, the fragmentation of benefits packages is unlikely to be compromising health system goals.

What does the evidence say about the health system impacts of benefit package fragmentation? There is little evidence exploring the plurality of benefits packages, especially in LMICs. Evidence from multiple contexts consistently demonstrates the negative impacts of a lack of access to high-quality comprehensive benefits packages on health outcomes and OOP payments,^58–63^ with clear implications for equity in health outcomes and financial risk protection. There is some evidence that high coverage, yet diverse benefits packages can contribute to achievement of health system goals. For example, Indonesia, Thailand and Vietnam have multiple benefits packages for different population segments (eg, the poor, formal workers, civil servants), with basic government-funded benefits packages for low-income groups. Despite lower coverage of services for low-income groups, the introduction of these packages (and reduction in fragmentation) has contributed to equity and improvements in access.^45^ However, there are downsides. Evidence from Colombia shows segmented benefits packages discourage integration of care, coordinated patient pathways and transfers of patients—especially for conditions that ‘straddle’ insurers and public providers.^64^ Large numbers of benefits packages (likely offered by private providers) can also contribute to confusion about what is covered or not, leading to inefficiencies and inequalities. Fragmentation of benefits packages also creates political barriers to expanding coverage as groups with generous packages fear their benefits will be diluted with a universal package. There may be some positives from multiple benefits packages. Having multiple benefits packages can allow tailored provision of services to different populations (eg, income groups or geographical regions), promoting system efficiency and reducing inequalities.^45^ Nonetheless, robust evidence analysing this is scant. Turkey is one example where multiple benefits packages were merged and unified (ie, reduced fragmentation), with evidence that this contributed to improved health outcomes, increased utilisation, better financial protection and improved satisfaction with the system.^37,65,66^ Overall system efficiency and effectiveness are not studied in relation to benefits package fragmentation.

### Provider fragmentation

Providers are the individuals or entities that deliver medical services, treatments and care to patients. They can be single health professionals or large organisations covering multiple levels of care (eg, community, primary and hospital care) with broad geographical coverage. A larger number of distinct providers in the system are indicative of higher fragmentation. This fragmentation may result in segments of the population accessing different providers or impose barriers in continuous care across providers. The number (and/or size) of providers is directly related to healthcare demand, but is dependent on other factors including local context, regulation and competition. For example, the local populations’ health needs and ability to pay are important determinants, as are local living standards which affect whether or not locations can attract sufficient health professionals. Regulation may establish public providers in certain areas or prohibit merging of providers.

The economics literature provides insights on provider behaviours—both public and private.^67,68^ Provider behaviours can be driven by competition, as multiple providers may compete for either contracts or patients or both. Greater provider fragmentation is likely indicative of increased competition, and competitive behaviours are likely to drive the wider health system impacts of provider fragmentation. However, this may not be the case in all settings. Even in areas with many providers, a few dominant providers, regulatory barriers to entry and/or exit, the significant bargaining power of large purchasers, or a lack of information for patients to compare or choose providers, may all stifle competition. Geography can also determine the levels of competition and fragmentation. For example, in sparsely populated regions the distances to providers (eg, primary care facilities or pharmacies) may be large, effectively meaning local populations rely on single providers. This means that many areas are likely to be locally unfragmented, even if there is more fragmentation in urban settings or nationally. Regulation may also prohibit competition in terms of price (either bidding for contracts from purchasers or fees charged to patients), quantity or quality. In these settings, providers may be serving distinct population segments, and market forces are unlikely to affect costs, quality or patient choice, with regulatory and administrative interventions required.

Many countries have either intentionally or unintentionally fostered, at least partly, competition within their health systems. Even in settings where prices are determined administratively, strategic interactions among providers and purchasers can affect the quantity and quality of services provided.^68^ On the one hand, competition may exert downward pressure on prices and/or lead hospitals to make higher effort levels to provide higher quality services to capture higher market shares. On the other hand, more concentrated markets (ie, less fragmentation), with fewer and larger providers, may result in efficiencies because of economies of scale, economies of scope and/or higher managerial skills.^69^

What are the health system impacts of provider fragmentation? In settings where there is low competition, we can make comparisons between different segmented providers. One example is vertical disease-oriented services, with evidence from sub-Saharan Africa showing these have led to duplication, inefficiencies and uncoordinated service activities.^34^ In systems with segmented public providers, for example, in Chile,^70^ Mexico,^71^ Colombia,^72^ we can compare different public providers, often with lower access and quality of care for government-funded providers (compared with private, employer or social security-financed providers). Comparing public and private systems in LMICs has shown private sector providers may have greater timeliness and better availability of professionals, medicines and equipment, although many private providers had poorer patient outcomes, lower efficiency due to unnecessary testing and treatment and greater financial barriers to access for low-income patients.^73^ Where patients do move between distinct providers, but they remain uncoordinated and there is little competition, fragmented care pathways can affect health system efficiency and care quality. In the USA, fragmented care provision is associated with increased utilisation of specialists with reductions in primary care utilisation.^74^ Interventions which reduce the fragmentation of public providers, for example, the merging of smaller hospitals, have been shown to increase efficiency.^75^

In markets that are both fragmented and competitive, the evidence remains unclear and mostly focused on hospitals in North America and Europe.^64,76^ Furthermore, there is a clear role for regulation in shaping how competitive (and fragmented) provider markets are. Evidence from Europe suggests competition can improve care quality, but the effects can be small and there may be no improvements in patient choice or efficiency.^77–79^ There is some evidence that introducing competition between hospitals can improve healthcare quality for some but not all conditions, and these improvements may be focused on the quality metrics reported to regulators.^39,77^ Providers may also select patients with cheaper diseases to increase profits (ie, cream-skimming) reducing system efficiency.^80–82^ In Georgia, a highly fragmented hospital market is associated with under-staffing, financial instability, wasteful marketing and demand-inducing behaviour.^83^ In settings where hospitals do not compete on prices, evidence suggests that a higher level of competition leads to a higher quality of healthcare services,^79,84,85^ but there impacts on quality are more ambiguous where the prices are determined through market interactions.^67,68^ Conversely, correlational evidence from the USA suggests that more concentration in hospital markets (ie, less competition) is associated with higher prices, while quasi-experimental evidence suggests that changes in the level of concentration stemming from hospital mergers have, on average, large impacts on prices.^68,86,87^ There is limited empirical evidence on competition and access, equity, health outcomes or financial risk protection.

### Payment fragmentation

Payments are the mechanisms by which purchasers (insurers, governments or patient paying OOP) pay providers to deliver health services. A multitude of mechanisms exist and these payments incentivise and disincentivise insurers and providers in multiple ways, for example, around populations covered, access and quality of care.^88^ These can be based on inputs (eg, facilities and staffing levels) or outputs (eg, services provided), or populations covered (eg, capitation). Pay for performance is increasingly being used to complement other payment flows and evidence suggests it can in some circumstances be associated with better processes of care (eg, access to care, screening, referral and health education).^89^

The number of different payment mechanisms between purchasers and providers is one indication of greater payment fragmentation. A high number of payment mechanisms is likely to introduce problems for coordination and effective service provision. There are likely administrative and bureaucratic inefficiencies for purchasers and providers in managing and adhering to payment obligations. Conversely, too few mechanisms will limit the scope to use payment mechanisms to govern and direct provider behaviours—for example, incentivising quality and efficiency. Importantly different types of payment mechanism are appropriate for different services—for example, hospital care, primary care and public health.^90^ A more effective measure of fragmentation in payments is the extent to which payments are used to increase overall coordination of the system—a focus not on the number of mechanisms, but the nature of the payments.

What are the health system impacts of payment fragmentation? Many systems have highly complex payment systems for healthcare, and evidence documents the negative impacts of fragmented (ie, uncoordinated) mechanisms. In settings where there are numerous uncoordinated payment mechanisms, for example, vertical disease programmes or separate public purchasers, this can lead to unclear incentives and signals to providers.^34^ Evidence from Brazil and Colombia demonstrates how fee-for-service discourages coordination of care.^64^ In Indonesia, despite consolidation of insurance schemes, primary care providers are paid through multiple streams for different services weakening coordination of the system.^32^ In the presence of different payment mechanisms, providers may have an interest in gaming the system, for example, shifting services to more favourable funding streams, which can lead to inefficiencies and inequities.^91^ Inefficiencies arise where multiple payers use different mechanisms for the same providers (eg, capitation vs fee-for-service) as there are conflicting incentives.

## Cross-cutting themes and interactions between the domains

### Regulation and decentralisation

Regulation and decentralisation are common themes across all the domains of fragmentation, mediating how fragmentation affects health system outcomes. Regulation is frequently used to counteract the negative impacts of fragmentation and promote coordination within the system.^32^ For example, this may be in the cross-subsidisation of risk-pools, the establishment of minimum benefit packages or the restriction to introduction of competition between providers. This may be because structural reform of the apparatus of the health system is not feasible nor desirable, and regulation is still desirable to steer the behaviour of actors even where fragmentation is low.

Much of the narrative and evidence on fragmentation has drawn on country-level evidence, but decentralisation remains a pervasive issue, especially in large countries. Many countries have sought to decentralise health system functions (including the financing, human resources management, organisation and delivery of services, regulations, planning and priority setting) to subnational levels of government, seeking to better align health resource allocation with the needs and preferences of local populations.^92^ There are different degrees of autonomy for subnational authorities with some units having broad decision-making powers over most functions and others with less, and these interact with fragmentation in different ways.^64,92,93^ Often, decentralisation fragments decision-making within the health system through the creation of new purchasing and provision structures at the subnational levels of government. Recent evidence suggests decentralisation can have more negative than positive impacts on the health system, particularly leadership, governance and financing and often exacerbating inequities.^94^ First, there is a need to consider health system fragmentation in subnational governments, in addition to national levels, for example, understanding local market conditions and devolved regulatory powers. Second, health system decentralisation interacts with various dimensions of fragmentation, often negatively. For example, state and federal governments may be both acting as purchasers, risk pools may be split across subnational geographies and geographically-delineated benefits packages and eligibility categories may exist. Furthermore, greater administrative capacity from lower levels of administration is required to regulate and coordinate the system, which might be affected by their lower levels of staff and capacity. Furthermore, the mechanisms of accountability of decision makers at the local levels make them accountable to both the desires of the beneficiary population and the supervision by the national authorities which may increase fragmentation of choices by local authorities.^95^

### Interactions between the fragmentation domains

It is challenging to provide a comprehensive overview of the interactions between the domains of financing fragmentation due to their inherent complexity. However, several general patterns can be identified. First, in the absence of strong regulation and coordination there can be amplification effects where different domains of fragmentation interact in ways that amplify their effects. For instance, when multiple fragmented payers contract with a fragmented provider market, the resulting inefficiencies, quality deficits and inequities are likely to be more severe than when only one of these domains is fragmented.

Second, there are coupling effects where certain domains are closely interrelated and frequently fragmented to a similar extent. This is particularly true for purchasing and risk pooling, which are often housed within the same institutions or closely affiliated agencies. Similarly, benefits packages, eligibility criteria and premium structures are tightly linked; fragmentation in one of these areas often facilitates or reinforces fragmentation in the others. Differentiated premiums and contributions may be used to define fragmented eligibility categories, each associated with distinct benefits packages. Fragmented payment systems also tend to coexist with fragmented provider markets. Third, there can be substitution effects where reducing fragmentation in one domain may partially offset fragmentation in others. For example, a single payer operating within a fragmented provider landscape may still help mitigate some of the negative consequences associated with broader system fragmentation.

## An overall understanding of health system financing fragmentation

The preceding sections have outlined how various health financing functions can be fragmented and summarised the mainly observational and context-specific evidence on the implications this has for overall health system performance. While it is difficult to determine which domains of fragmentation are most consequential, their effects are often interdependent. In some cases, high fragmentation in one domain may be partially mitigated by greater integration in another. Moreover, not all forms of fragmentation are inherently detrimental. For instance, competition among providers, strategic use of diverse payment mechanisms, and variation in benefits packages can—if well-regulated—enhance responsiveness, innovation, and consumer choice.

Nonetheless, some overarching observations can be made:

- Fragmentation is a pervasive feature of health systems globally. Drawing on cross-country descriptions and a small number of comparative studies, extreme fragmentation is frequently associated with negative outcomes—particularly in terms of equity, financial risk protection and health outcomes. However, causal effects are difficult to establish. Economic theory and some case studies suggest that some degree of fragmentation may offer benefits such as flexibility, adaptability and tailored service delivery.
- Strengthening universal health coverage (UHC) likely necessitates a reduction in fragmentation. UHC relies heavily on public financing mechanisms (eg, taxation or social health insurance). Based on theoretical groups and some comparative empirical evidence, high levels of fragmentation are likely to undermine efforts to achieve equitable access to high-quality services, by precluding broad population coverage, comprehensive benefit packages and low financial hardship for all.
- The mere presence of multiple entities is not a sufficient indicator of harmful fragmentation. A more meaningful assessment must consider the degree of coordination among these entities and their relative size and influence within the system.
- Regulation is a critical tool for managing fragmentation. Theory and some case studies suggest effective governance can mitigate the adverse effects of fragmentation by aligning incentives, standardising practices and ensuring accountability across actors.
- Decentralisation often contributes to fragmentation. While decentralisation can enhance local responsiveness and innovation, it must be carefully balanced with mechanisms that promote system-wide coordination, efficiency and equity.
- Legacy structures and entrenched stakeholder interests limit policy-maker options for reforming fragmented health systems. Current health system fragmentation stems from historical infrastructure, policies and reforms, and possible efforts to reduce fragmentation and improve health system performance will likely be constrained.
- Coordination in less fragmented systems is not without cost. Information asymmetries, administrative burdens, and transaction costs can limit the effectiveness of centralised governance. These coordination frictions may justify a degree of decentralisation or functional fragmentation, particularly in contexts where local responsiveness, innovation or stakeholder engagement are essential.^96^ Moreover, such costs may present substantial short-term barriers to integration, even when long-term benefits are anticipated.

### Limitations

This review has several limitations. Our approach to identifying studies was not fully exhaustive or systematic and we may have missed relevant studies. We searched for specific phrases, but may have missed other theories and alternative perspectives from other fields using different terms. The literature we identified is heavily skewed towards high-income countries, reflecting underlying publication and research funding biases. Fragmentation across financing domains typically occurs simultaneously, making it challenging to isolate the specific contribution of individual domains (eg, purchasers vs risk pools or payment mechanisms) to observed outcomes. It was not feasible to undertake a formal risk-of-bias or quality assessment of included studies, and the empirical work we summarise is predominantly observational, limiting the strength of causal inference about fragmentation and health system outcomes.

## Conclusions

Addressing and managing fragmentation of health systems financing presents a major challenge for countries striving to achieve UHC. This review outlines how health system financing fragmentation can be conceptualised as insufficient coordination within and between six domains. Across settings, the available empirical literature (which is dominated by observational studies from high-income countries and a smaller set of LMIC case studies) generally finds that high fragmentation is associated with adverse outcomes in terms of health system performance, including increased costs, inefficiencies and inequities—particularly for disadvantaged populations reliant on underfunded public services or unregulated private providers. However, the direction and magnitude of these associations vary by context, and there is limited robust comparative evidence that can isolate the causal effects of fragmentation from other features of health systems. Yet fragmentation is not uniformly harmful. In specific contexts and under strong governance, theory and some descriptive case studies suggest regulated pluralism may support UHC objectives to enhance responsiveness, spur innovation and expand choice. Under certain conditions, financing fragmentation may enable responsiveness to local needs, fostering regulated competition to improve quality and efficiency, promoting individual choice and enhancing overall societal welfare.

Across the six domains, three broad types of evidence underpin our conclusions: (1) a small number of robust comparative empirical analyses (eg, on purchaser consolidation, risk pooling and competition among providers); (2) context-specific case studies and reform evaluations from particular countries and (3) economic theory and logic, which we use to interpret observed patterns and hypothesise potential mechanisms where direct data are lacking. Notably, nearly all evidence from LMICs is from groups (2) and (3). Future research should prioritise comparative, longitudinal and quasi-experimental studies, especially from LMICs, that can more rigorously estimate the causal impacts of different fragmentation patterns and evaluate the effects of concrete reform options on equity, efficiency and financial protection.

The complexity of health system financing fragmentation, including the dynamic interactions among different domains of fragmentation, calls for more nuanced, empirical investigation. This includes better measures of fragmentation, quantification of trade-offs and evaluations of reforms that affect health system fragmentation and coordination. Context-specific assessments are important given the trade-offs between positives and negatives that vary across settings. For policymakers and reformers, the priority should not necessarily be to eliminate all fragmentation, but rather to strategically manage it. The framework can be used as a diagnostic tool to map existing financing arrangements across the six domains, identify where fragmentation is most problematic and prioritise concrete options such as consolidating risk pools, harmonising benefit packages or simplifying payment mechanisms. Our synthesis also underscores that financing reforms which ignore existing fragmentation patterns risk unintended increases in inequity and system costs, whereas carefully sequenced consolidation of purchasers and risk pools, coupled with strong regulation, offers a more credible route to advancing UHC. Strengthening coordination mechanisms, improving regulatory oversight and aligning incentives across actors are essential steps towards mitigating the negative effects of fragmentation while preserving potential benefits. In this regard, coordination should be viewed not only as a remedy to fragmentation but as a core function of effective health system governance.

## Data Availability

This research uses published articles and literature. No new data were generated or analysed in support of this research.
